# Associated Physicians and Surgeons of Santa Clara Valley

**Published:** 1897-02

**Authors:** 


### Associated Physicians and Surgeons of Santa Clara Valley.

SAN JOSE, CAL., November 18, 1896.

*Editors Texas Medical Journal:*

DEAR SIRS:—We ask you to give publicity to this letter and accompanying resolutions, to the end that in all communities afflicted with the pestiferous practice of lodge doctoring, physicians may be encouraged to assert their independance through organization.

Here, in Santa Clara county, Cal., containing 70,000 population, all the physicians of the county, numbering 124, have entered the compact that has ridden us of a slavish evil, and wrought independence and freedom for the practitioners of medicine. Investigation shows that medical compensation for lodge work averages about 15 cents on the dollar.

Even respectable lodge physicians feel a sense of degredation in giving their services for 15 cents on the dollar, and the ever-increasing spread of these alleged charitable institutions is absolutely destructive to the business of other physicians.

The main incentive of the persons who band themselves together in lodges is to get cheap doctoring; they are willing to take, but not to give. They belong to protective unions, and the same right should not be denied physicians. Ninety-nine per cent. of these people are able to pay reasonable fees to physicians, but will not do so as long as a few doctors in every community, for the sake of immediate gain, can be induced to stand as driven guys to the lodge politicians. No preacher or lawyer would give his services to these people for 15 cents on the dollar. No grocery store or merchandise firm would contract to supply these lodges with goods at 15 cents on the dollar for actual worth.

The remedy indicated in the subjoined resolution is simple, and manifestly efficacious, depending upon the personal honor and free will of those concerned. Where one doctor temporarily profits by contract work the business and ethical rights of fifty others are violated; hence an overwhelming *esprit de corps* is created among physicians which will sustain a strict observance of the pledge.

LINCOLN COTHRAN, M. D., Secretary.

N. B.--Please send a sample copy of journal containing article to the secretary.

RESOLUTIONS ADOPTED BY THE PHYSICIANS OF SANTA CLARE COUNTY, CAL.

WHEREAS, Rendering professional services at a stipulated fee per capita per annum is derogatory to the dignity of the medical profession, we, the undersigned physicians and surgeons of Santa Clara county, California, enter into the following agreement:

First.—We mutually, jointly, and individually, pledge our word of honor not to enter into any contract or agreement, or renew any existing contract or agreement, either written, verbal or implied, to render medical or surgical services to any lodge, society, association or organization.

Second.—We will not render medical or surgical services to the members of the above mentioned bodies for less compensation than we charge the general public for similar services.

Third.—This agreement shall not be construed to affect existing contracts between physicians and surgeons and the above mentioned bodies.

Fourth.—These pledges shall take effect and be in force for a term of three (3) years from and after May 22, 1896.

This agreement shall not apply to hospitals and purely public charitable institutions.

*San Jose.*—Leonard Pratt, J. K. Secord, Lincoln Cothran, J. E. Trueman, H. W. Felton, W. T. Hicks, J. F. Burns, J. U. Hall, Sr., Fred H. Bangs, Anna Flynn, J. R. Curnow, O. W. Yeargain, Chauncey R. Burr, W. D. McDougall, E. Wislocki, H. B. Gates, W. B. Hill, W. K. Davis, C. E. Hailstone, J. Turner, Elizabeth R. Osborn, W. A. Gordon, C. Y. Brownlee, C. E. Hablutzel, Carrie H. Goss, J. S. Potts, A. Jayet, H. C. Brown, Drs. A. and J. McMahon, A. B. Bishop, Jacob N. Brown, Robt. Caldwell, M. A. Southworth, T. A. Perin, F. La Spada, E. Filipello, J. N. Johnston, Geo. W. Seifert, J. Underwood Hall, Jr., J. L. Assay, P. M. Lusson, Thos. Kelley, H. J. B. Wright, Philip Miller, Geo. D. Brownlee, Charles H. Walter, A. C. Simonton, Wm. Simpson, Elizabeth Gallimore, E. K. Macomber, R. H. Burke, J. T. Harris, J. D. Grissim, Fred Gerlach, Wm. E. Keith, C. A. Wayland, R. E. Pierce, P. G. Denninger, W. N. Williams, Irwin N. Frasse, Ed Ulrich, Jno. J. Miller, W. T. McNary, Graily H. Hall, F. B. Eaton, Ralph T. Orvis, Henry Case, B. A. Guyton, D. H. Johnson, Sarah B. Bailey, Florence Belknap, C. A. E. Hertel, C. K. Farley, Scott P. Woodin, Mrs. J. M. Bowen, Wallace E. Parkman, J. D. Meng, W. H. Crothers, R. W. M. Payne.

*Santa Clara.*—S. M. Dodson, H. H. Warburton, D. A. Beattie, E. H. Smith, H. F. Carpenter, C. E. Adams, Geo. W. J. Fowler, J. W. Paul.

*Los Gatos.*—R. A. Urquhart, R. P. Gober, S. Grant Moore, J. W. Walker, F. W. Knowles, R. E. Freeman, Sarah H. Graves, T. A. A. Belinge, Mrs. Mary B. Mallory, W. T. Fretwell.

*Palo Alto.*—Harriet F. Pillsbury, W. L. Adams, Frank H. Moss, E. M. Price, W. H. Kellogg, E. W. Charles.

*Mayfield.*—F. E. Buck.

*Mt. View.*—D. D. Johnson, H. B. Emerson, C. O. Gates.

*Morgan Hill.*—J. T. Higgins.

*Gilroy.*—J. W. Thayer, C. C. J. Wachendorf, H. R. Chesbro, Jonas Clark, H. S. Scott.

*Saratoga.*—J. M. Whipple, R. L. Hogg.

*Agnews.*—W. F. Pratt, L. E. Stocking, F. W. Hatch.

*Campbell.*—M. J. Gates, Chas. N. Cooper.

*Milpitas.*—W. L. Wilson, F. M. Peironnet.

*College Park.*—Daniel Kienborts.

STANDING COMMITTEE.

Geo. W. J. Fowler, Thos. Kelley, Ed Ulrich, H. C. Brown, R. A. Urquhart, Geo. W. Seifert, Frank H. Moss, C. E. Hailstone, R. P. Gober, H. J. B. Wright, J. W. Paul, W. D. McDougall, C. E. Wayland, A. Jayet, Chairman; Lincoln Cothran, Secretary.

The duty of the Standing Committee shall be to interview new coming physicians, etc., and, in general, to carry out the purposes of the agreement. Communications may be addressed to the Chairman or Secretary.

---

NEW YORK CITY, Jan. 8. 1896.

*Editor Texas Medical Journal:*

MY DEAR SIR:—Will you kindly inform me at your earliest convenience if there is a State Board of Examination in your State for an Eastern physician to pass. I am a graduate from a Western college, and would like to come to your State. An early answer will oblige.

Yours very truly,

SAMUEL S. M., M. D.

311 E. 72d st., New York City, N. Y.

[We wrote him that there is no State Board of Examination in Texas, but that there is a district board in each district; but whether for an "Eastern physician to pass," we couldn't say—thought he might possibly fail. We were under the impression, we told him, that no discrimination is made by these boards between "Eastern" physicians and physicians from any other old place, and suggested that perhaps a Western physician with a diploma from the East stood about as good a chance in Texas as an Eastern physician with a diploma from the West.]

